# Metabolic Profiling Predicts Response to Anti–Tumor Necrosis Factor α Therapy in Patients With Rheumatoid Arthritis

**DOI:** 10.1002/art.37921

**Published:** 2013-05-30

**Authors:** Sabrina R Kapoor, Andrew Filer, Martin A Fitzpatrick, Benjamin A Fisher, Peter C Taylor, Christopher D Buckley, Iain B McInnes, Karim Raza, Stephen P Young

**Affiliations:** 1 University of Birmingham and the Sandwell and West Birmingham Hospitals NHS TrustBirmingham, UK; 2 University of Birmingham and the University Hospitals Birmingham NHS Foundation TrustBirmingham, UK; 3 University of BirminghamBirmingham, UK; 4 Kennedy Institute of Rheumatology and Imperial CollegeLondon, UK; 5 Kennedy Institute of Rheumatology, University of OxfordLondon, UK; 6 Institute of Infection, Immunity, and Inflammation and University of GlasgowGlasgow, UK

## Abstract

**Objective:**

Anti–tumor necrosis factor (anti-TNF) therapies are highly effective in rheumatoid arthritis (RA) and psoriatic arthritis (PsA), but a significant number of patients exhibit only a partial or no therapeutic response. Inflammation alters local and systemic metabolism, and TNF plays a role in this. We undertook this study to determine if the patient's metabolic fingerprint prior to therapy could predict responses to anti-TNF agents.

**Methods:**

Urine was collected from 16 RA patients and 20 PsA patients before and during therapy with infliximab or etanercept. Urine metabolic profiles were assessed using nuclear magnetic resonance spectroscopy. Discriminating metabolites were identified, and the relationship between metabolic profiles and clinical outcomes was assessed.

**Results:**

Baseline urine metabolic profiles discriminated between RA patients who did or did not have a good response to anti-TNF therapy according to European League Against Rheumatism criteria, with a sensitivity of 88.9% and a specificity of 85.7%, with several metabolites contributing (in particular histamine, glutamine, xanthurenic acid, and ethanolamine). There was a correlation between baseline metabolic profiles and the magnitude of change in the Disease Activity Score in 28 joints from baseline to 12 months in RA patients (*P* = 0.04). In both RA and PsA, urinary metabolic profiles changed between baseline and 12 weeks of anti-TNF therapy. Within the responders, urinary metabolite changes distinguished between etanercept and infliximab treatment.

**Conclusion:**

The clear relationship between urine metabolic profiles of RA patients at baseline and their response to anti-TNF therapy may allow development of novel approaches to the optimization of therapy. Differences in metabolic profiles during treatment with infliximab and etanercept in RA and PsA may reflect distinct mechanisms of action.

The introduction of anti–tumor necrosis factor α (anti-TNFα) treatment has revolutionized the management of rheumatoid arthritis (RA) (1–4). Several agents are available within this class, but response rates are imperfect; only 26–42% of patients achieve a good European League Against Rheumatism (EULAR) response (5) within 6 months (6–8). Given the high cost of these therapies and implications for disease progression in nonresponders waiting 3–6 months for clinical reassessment, the ability to predict treatment responses at baseline is an important goal.

The etiology of RA is not fully understood but involves both genetic and environmental factors. In addition to synovitis there are widespread systemic effects mediated by proinflammatory cytokines that affect metabolism. Muscle wasting is a common feature of RA and its extent is associated with RA disease activity (9), but low body mass index is uncommon as fat mass is preserved or even increased (10). The extent of the metabolic changes and the types of metabolites seen may therefore be good markers of cytokine-mediated inflammatory processes in RA.

Several studies have used metabolomic analysis in patients and animal models of inflammatory disease (11–15). Given the integrated nature of systemic metabolism, the analysis of multiple metabolites may provide a better understanding of the disease-associated changes. Metabolomic analysis, based on nuclear magnetic resonance (NMR) spectroscopy of biofluids, can be used to identify a broad range of metabolites simultaneously. Using this approach, the identification of several metabolites in cancer and cardiovascular disease has provided insights into disease mechanisms and has highlighted their potential as biomarkers of disease activity and response to therapy (16–18).

Systemic changes in many low molecular weight metabolites are reflected by their levels in urine, and, indeed, metabolomic analysis of urine samples has been used in inflammatory conditions such as inflammatory bowel disease (IBD) (19–21), to successfully distinguish different types of IBD, and to identify the presence of ongoing intestinal inflammation. Metabolomic profiles have also been shown to be altered during therapy (16). Consequently, we sought to assess whether metabolomic profiles in the urine may have a role in predicting responses to TNF antagonists in patients with RA and psoriatic arthritis (PsA).

## PATIENTS AND METHODS

### Patients

Patients were part of a multicenter study (Glasgow Royal Infirmary [PsA patients only], Queen Elizabeth Hospital, Birmingham [PsA patients only], and Charing Cross Hospital, London [RA patients only]) comparing responses to infliximab and etanercept. All patients were age ≥18 years. RA patients were required to fulfill the 1987 revised classification criteria of the American College of Rheumatology (22), to be positive for rheumatoid factor (RF) and/or anti–cyclic citrullinated peptide (anti-CCP) antibodies, and to have a disease duration of ≥6 months and a Disease Activity Score in 28 joints (DAS28) of ≥4.0 (23). The PsA patients were required to have psoriasis at screening, ≥3 swollen and ≥3 tender peripheral joints, negativity for RF and anti-CCP antibodies, and a disease duration of ≥6 months. Treatment with at least 1 disease-modifying antirheumatic drug (DMARD) had failed for all patients, and all patients were treated with methotrexate at a dose of at least 7.5 mg weekly, stable for at least 4 weeks prior to commencing anti-TNFα therapy. No other DMARDs were allowed within the 4 weeks prior to commencing treatment, but prednisolone was allowed provided the dose remained stable and did not exceed 10 mg daily.

Participants (16 RA patients and 20 PsA patients) were randomly assigned to receive 3 mg/kg infliximab at weeks 0, 2, and 6 and then every 8 weeks until week 46, or to receive 25 mg etanercept twice weekly for 52 weeks. Therapy was kept stable for the first 3 months. After 3 months, therapy could be changed as required, including escalation of methotrexate therapy to 25 mg weekly in apparent nonresponders. Clinical data, including erythrocyte sedimentation rate, DAS28, and Health Assessment Questionnaire scores (24), were collected at baseline and monthly up to week 52. A good clinical response in RA was defined as a DAS28 of ≤3.2 and improvement in the DAS28 of >1.2 after therapy (25). A good response in PsA was defined as an improvement in 2 factors (with at least 1 being a joint count) with worsening in none of the following 4 factors: patient's global assessment of disease activity, physician's global assessment of disease activity, tender joint count, and swollen joint count (26). Random urine samples were collected from the patients at baseline and at 12 weeks and were snap-frozen and stored at –80°C. The study was conducted in compliance with the Declaration of Helsinki, and ethical approval was obtained from the West Glasgow Ethics Committee. All subjects gave written informed consent.

### Metabolomic analysis

After thawing, urine samples (1 ml) were centrifuged at 13,000*g* for 5 minutes, and samples were prepared using a standard protocol (27). Briefly, urine was buffered with phosphate buffer (100 m*M*), brought to concentrations of 10% D_2_O and 0.5 m*M* TMSP, and the pH was adjusted to 7.0 twice over 30 minutes. The sample was then centrifuged and loaded into a standard 5-mm NMR tube for spectroscopy.

One-dimensional (1-D) ^1^H spectra were acquired at 300°K using a standard spin-echo pulse sequence with water suppression using excitation sculpting on a Bruker DRX 500 MHz NMR spectrometer equipped with a cryoprobe. Samples were processed and data were calibrated with respect to the TMSP signal. Spectra were read into Prometab (28) (custom written software in MatLab 7; MathWorks) and were truncated to a range of 0.8–10.0 parts per million. Spectra were segmented into 0.005 ppm (2.5 Hz) chemical shift “bins,” and the spectral areas within each bin were integrated. Spectra were corrected for baseline offset and then normalized to a total spectral area of unity, and a generalized log transformation was applied (28). Binned data were then compiled into a matrix, with each row representing an individual sample.

### Measurement of metabolites

Glutamine levels were measured in the urine samples using high-performance ion-exchange chromatography. Xanthurenic acid levels were measured using a fluorometric method (29).

### Statistical analysis

The data bins from groups of spectra were mean-centered and then assessed using 3 techniques. First, partial least-squares discriminant analysis (PLS-DA) was used to perform supervised clustering of samples using PLS_Toolbox (version 5.8; Eigenvector Research) in MatLab (release 2009a). PLS-DA was cross-validated using Venetian blinds (30), a method that reassigns randomly selected blocks of data to the PLS-DA model to determine the accuracy of the model in correctly assigning class membership. Second, GALGO, a package available in the statistical environment R, was used to further model the relationship between good responders and those who did not respond well using a genetic algorithm search procedure coupled to statistical modeling methods for supervised classification (31). The results of GALGO analyses are presented as principal components analysis (PCA) plots, where the x- and y-axes represent first and second principal components providing the greatest variation between samples and the next largest unrelated variation, respectively. GALGO analysis was cross-validated using K-fold cross-validation, where the original sample is randomly partitioned into subsamples and each observation is used for both training and validation. Finally, we used partial least-squares regression, a regression method that identifies which metabolites can predict a continuous variable. This analysis yields r^2^, a measure of the goodness-of-fit of the linear regression, while permutation testing assessed the significance of this prediction.

Lists of metabolites providing the greatest discrimination between groups were then identified for each technique. Using multivariate analyses, peaks with large weightings were identified from the PLS-DA weightings plot. Metabolites were identified using these peaks. GALGO analysis produces a list of “bins” of ranked importance which contribute to the separation between the groups. The partial least-squares regression model represents the 90 “bins” or regions of the spectra which had the greatest influence on the correlation with the change in DAS28. These bins were used to identify the discriminatory metabolites. An NMR database (Human Metabolome Database version 2.5) and Chenomx NMR suite were used to identify the metabolites.

## RESULTS

### Prediction of response to anti-TNF therapy

After 12 months of anti-TNF therapy, RA patients were divided into 2 groups according to their response, as determined by EULAR criteria (Table 1). Response to anti-TNF therapy was also assessed at 3 months, but only 4 patients had a good response (as determined by EULAR criteria) at this stage. Only 1 PsA patient did not respond to treatment with a TNF antagonist according to the predefined response criteria; it was therefore not possible to look at prediction of response in PsA.

**Table 1:** Baseline characteristics of rheumatoid arthritis patients, by response to anti-TNF therapy at 12 months*

	Good response to TNF antagonists (n = 7)	Not good response to TNF antagonists (n = 9)†
Age, years	50.0 ± 13.40	52.67 ± 12.83
Female, no. (%)	7 (100)	9 (100)
BMI, kg/m^2^	26.16 ± 3.75	26.97 ± 5.64
Prednisolone, no. receiving	1	2
NSAIDs, no. receiving	4	4
Baseline methotrexate dose, mg/week	13.57 ± 4.76	15.83 ± 6.12
DAS28	6.041 ± 1.06	6.46 ± 0.91
CRP, mg/ml	21.31 ± 15.97	7.02 ± 7.97
RF positive, no.	6	8
Anti-CCP antibody positive, no.	6	8
Urinary albumin-to-creatinine ratio	1.60 ± 2.71	0.29 ± 0.45

* Except where indicated otherwise, values are the mean ± SD. Except for the C-reactive protein (CRP) level (*P* = 0.03 by unpaired *t*-test), there were no significant differences between the groups. Anti-TNF = anti–tumor necrosis factor; BMI = body mass index; NSAIDs = nonsteroidal antiinflammatory drugs; DAS28 = Disease Activity Score in 28 joints; RF = rheumatoid factor; anti-CCP = anti–cyclic citrullinated peptide.

† Corresponds to moderate and poor response, as determined by European League Against Rheumatism criteria.

**Figure 1:**
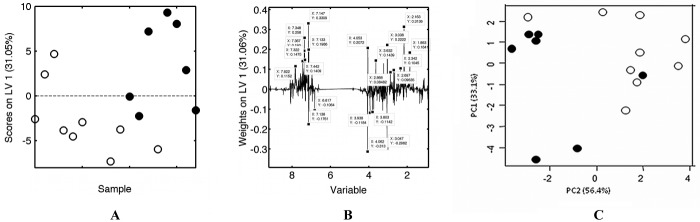
Metabolic fingerprinting distinguishes between baseline urine samples from rheumatoid arthritis (RA) patients who have good responses to tumor necrosis factor (TNF) antagonists at 12 months and those who do not. **A,** One-dimensional ^1^H nuclear magnetic resonance (NMR) spectra of baseline urine samples from RA patients with (solid circles) or without (open circles) a good response to TNF antagonists at 12 months were subjected to supervised analysis (partial least-squares discriminant analysis [PLS-DA]). The sample number is plotted against the score in (contribution to) latent variable 1 (LV1), and the percent value on the y-axis shows the proportion of the total variance in the data captured by LV1 in the PLS-DA model. The horizontal dashed line at zero segregates samples with positive and negative scores. **B,** Weightings plot of the PLS-DA model of spectral data from baseline urine samples from the RA patients who did or did not have a good response at 12 months highlights major regions of the spectra that distinguish between the sample groups. The major discriminating peaks are labeled with their chemical shift (in parts per million) on the x-axis and LV weighting on the y-axis. The percent value on the y-axis indicates the proportion of the total variance in the data captured by LV1. **C,** One-dimensional ^1^H NMR spectra of baseline urine samples from RA patients with (solid circles) or without (open circles) a good response to TNF antagonists at 12 months were subjected to principal components analysis using GALGO analysis. The percent values on the x and y axes indicate the proportion of the variance captured by each principal component (PC1 and PC2).

NMR spectra of stored baseline urine samples were acquired and analyzed in order to identify differences between the 2 groups. Supervised PLS-DA analysis (Figure 1A) showed a clear distinction between patient groups segregated according to clinical response. This model distinguished samples with or without a good response with a sensitivity of 66.7% and a specificity of 57.1%. A weightings plot, which indicates regions of the NMR spectra that contribute to this separation (Figure 1B), was used to identify the discriminatory metabolites responsible for the difference in response, and these are shown in Table 2.

**Table 2:** Baseline urinary metabolites most strongly correlated with response to anti-TNF therapy using partial least-squares regression, GALGO, and PLS-DA models*

Rank†	Metabolite (chemical shift[s], in ppm), PLS-DA model‡	VIP value§	Metabolite (chemical shift[s], in ppm), GALGO model	Metabolite (chemical shift[s], in ppm), partial least-squares regression model
1	↑Histamine (7.15)¶	91.54	Trimethylamine (2.87)	Citrate (2.67, 2.53, 2.56)
2	↑Glutamine (2.16, 2.34)¶	87.72	Thiamine (7.41)	Phosphocreatinine (3.04, 3.94)#
3	↓p-hydroxyphenylpyruvic acid (4.06, 7.14, 6.82, 7.12)#	72.72	Histamine (7.24, 8.01, 8.00, 7.12)¶	Ethanolamine (3.16, 3.74)¶
4	↓Phosphocreatine (3.05, 3.94)#	60.58	Thymine (7.37, 7.35, 1.86)#	Creatinine (4.05, 4.06, 3.05)#
5	↑Thymine (7.35, 1.86)#	61.8	Ethanolamine (3.17, 3.80)¶	Histamine (7.13, 3.28, 7.15)¶
6	↑Creatinine (3.04, 4.05)#	50.58	Phenylacetic acid (7.29, 7.39, 7.30)#	Glutamine (2.16)¶
7	↑Xanthurenic acid (7.37, 7.13)¶	36.71	Glutamine (2.12, 2.13)¶	p-hydroxyphenylpyruvic acid (6.82, 6.8225, 4.06, 7.12)#
8	↑Phenylacetic acid (3.63, 7.44, 7.32)#	21.48	Xanthurenic acid (6.96)¶	Dimethylamine (2.70)
9	↑Xanthine (7.82)#	12.68	Xanthine (7.90)#	Xanthurenic acid (7.15)¶
10	↓Ethanolamine (3.80)¶	7.59	Tartaric acid (4.38)	–
11	–	–	3-phosphoglyceric acid (4.14)	–

* The top 20 bins (partial least-squares regression and GALGO models) and peaks (partial least-squares discriminant analysis [PLS-DA] model) were identified; the metabolites these represent and their corresponding chemical shifts in parts per million are shown.

† Extent of contribution to differentiation between responders and nonresponders to anti–tumor necrosis factor (anti-TNF) agents.

‡ ↑ indicates up-regulation of metabolites in urine samples from patients who had a good response to TNF antagonists. ↓ indicates down-regulation of metabolites in urine samples from patients who had a good response to TNF antagonists.

§ Variable importance of the projection (VIP) values were calculated using PLS-DA.

¶ Identified by all 3 methods.

# Identified by 2 different methods.

The partial least-squares regression model represents the 90 “bins” or regions of the spectra that had the greatest influence on the correlation with the change in DAS28. The GALGO model identifies the bins that have the greatest influence on the separation. For the PLS-DA model, the metabolites were identified from the weightings plot, which indicates regions of the NMR spectra that contribute to the separation. The top 20 bins were identified using GALGO and partial least-squares regression and the metabolites identified from these 20 bins. From the PLS-DA weightings plot the top 20 peaks were identified, and the metabolites were identified from these.

GALGO analysis was then used to reanalyze the data, first in order to verify the results obtained using a further supervised analysis technique, and second to use the superior modeling power of the GALGO genetic algorithm, which removes irrelevant variables more effectively. The PCA plot yielded by GALGO analysis showed a clear distinction between RA patients segregated according to clinical response (Figure 1C). The cross-validation of this model was shown to distinguish samples from patients who would not have a good response and samples from patients who would have a good response with a greatly improved sensitivity of 88.9% and specificity of 85.7%. GALGO analysis was further used to identify the discriminatory metabolites responsible for the difference in response as shown in Table 2.

Finally, the relationship between baseline metabolite profiles and the change in DAS28 over 12 months was assessed using partial least-squares regression. This analysis was repeated 100 times with and without randomization of the NMR bin data. There was a significant association between the change in DAS28 and baseline RA metabolites (*P* = 0.04). Permutation testing with 90 NMR bins included (as optimized by forward selection) demonstrated that the regression model was statistically valid (*P* < 0.01).

There was a significant difference between the C-reactive protein (CRP) level of patients who responded to TNF antagonists and the CRP level of those who did not respond (*P* = 0.03). We therefore used partial least-squares regression to further analyze the relationship between CRP and baseline metabolites in order to investigate potential confounding variables; this did not reveal any significant association (*P* = 0.52), suggesting that the difference we found was independent of the inflammatory processes reflected in the CRP levels. Grouping the metabolite data into quartiles according to the CRP values also failed to separate patient groups on PCA or PLS-DA (data not shown). Previous studies have shown that patients with RA have subclinical nephropathy (32,33) and that the urinary albumin-to-creatinine ratio is a sensitive marker of disease activity in RA (32). We measured the albumin-to-creatinine ratio in the urine samples, and there was no significant difference in this ratio between patients who responded to TNF antagonists and those who did not (*P* = 0.17). We also performed regression analysis for metabolic profiles at baseline against the albumin-to-creatinine ratio, and this was not significant (*P* = 0.31), suggesting that the relationship we found between baseline urinary metabolic profiles and the DAS28 was independent of microalbuminuria.

### Comparison of metabolites predicting response to therapy in RA

Metabolites that were associated with a change in the DAS28 are shown in Table 2. The metabolites histamine, glutamine, xanthurenic acid, and ethanolamine were identified by all 3 analytic methods. Furthermore, several metabolites were identified by at least 2 of the 3 different methods, including p-hydroxyphenylpyruvic acid, phosphocreatine, thymine, creatinine, phenylacetic acid, and xanthine. These findings cross-validate the analyses used. We were also able to identify glutamine and xanthurenic acid in the urine samples that were used for NMR analysis using ion-exchange chromatography and a fluorometric method, respectively. There was a good correlation between the NMR peak heights and the assayed levels of xanthurenic acid (r = 0.73 [Spearman's correlation coefficient], *P* = 0.001) and a strong trend in the results for glutamine (r = 0.46 [Spearman's correlation coefficient], *P* = 0.07), which helped to validate our interpretation of the NMR data. However, the assayed levels of glutamine and xanthurenic acid were not significantly higher in the urine samples from the patients who had a good response, which suggests that while these individual metabolites contribute strongly to the discrimination, the whole set of metabolites present in the fingerprints is needed to fully separate the groups.

### Effect of TNFα antagonists on metabolite profiles

**Figure 2:**
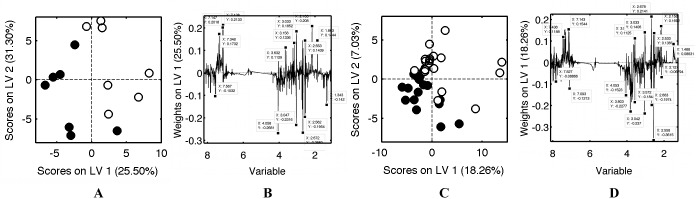
Metabolic fingerprinting enables identification of metabolites that are altered after treatment with TNF antagonists in patients with a good response. **A,** One-dimensional ^1^H NMR spectra of urine samples obtained at baseline (open circles) and 12 weeks (solid circles) from RA patients with a good response to TNF antagonists at 12 months were subjected to supervised analysis (PLS-DA). The score in LV1 is plotted against the score in LV2, and the percent values on the x and y axes show the proportions of the total variance in the data captured by LV1 and LV2 in the PLS-DA model. The horizontal dashed lines at zero segregate samples with positive and negative scores. **B,** Weightings plot of the PLS-DA model of spectral data from urine samples obtained from RA patients who responded to TNF antagonists highlights major regions of the spectra that distinguish between the baseline and 12-week samples. The major discriminating peaks are labeled with their chemical shift (in parts per million) on the x-axis and LV weighting on the y-axis. The percent value on the y-axis indicates the proportion of the total variance in the data captured by LV1. **C,** One-dimensional ^1^H NMR spectra of urine samples obtained at baseline (open circles) and 12 weeks (solid circles) from psoriatic arthritis (PsA) patients with a good response to TNF antagonists at 12 months were subjected to supervised analysis (PLS-DA). The score in LV1 is plotted against the score in LV2, and the percent values on the x and y axes show the proportions of the total variance in the data captured by LV1 and LV2 in the PLS-DA model. The horizontal dashed lines at zero segregate samples with positive and negative scores. **D,** Weightings plot of the PLS-DA model of spectral data from urine samples from PsA patients who responded to TNF antagonists highlights major regions of the spectra that distinguish between the baseline and 12-week samples. The major discriminating peaks are labeled with their chemical shift (in parts per million) on the x-axis and LV weighting on the y-axis. The percent value on the y-axis indicates the proportion of the total variance in the data captured by LV1. See Figure 1 for other definitions.

The details of the patients receiving etanercept and infliximab are shown in Table 3. We investigated the effect of anti-TNF therapy on metabolic profiles longitudinally, comparing baseline and 12-week (during therapy) urine samples using supervised PLS-DA analysis (sensitivity of 71.4% and specificity of 57.1% in RA and sensitivity and specificity of 61.1% in PsA) and GALGO analysis (sensitivity of 100% and specificity of 82.9% in RA and sensitivity of 71.8% and specificity of 69.5% in PsA). Using the weightings plot, we determined that in patients with RA who responded to TNF antagonists there were high levels of glutamine, phenylacetic acid, and histamine in the baseline urine samples and higher levels of methylamine and creatinine in the 12-week urine samples. Similar changes in metabolites were also seen in the urine samples from the patients with PsA who responded to TNF antagonists (Figure 2).

**Table 3:** Characteristics of the rheumatoid arthritis and psoriatic arthritis patients, by treatment group*

	Patients receiving infliximab (n = 18)	Patients receiving etanercept (n = 16)
Age, years	48.6 ± 10.60	47.46 ± 14.78
Female, no. (%)	13 (72)	13 (81)
BMI, kg/m^2^	28.43 ± 4.56	28.36 ± 9.33
Prednisolone, no. receiving	6	3
NSAIDs, no. receiving	10	9
DAS28 at baseline†	6.22 ± 0.95	6.35 ± 1.05

* Except where indicated otherwise, values are the mean ± SD. See Table 1 for definitions.

† Rheumatoid arthritis patients only.

**Figure 3:**
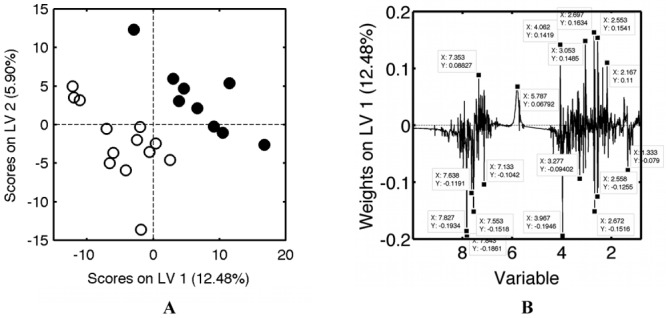
Metabolic fingerprinting of urine samples from RA patients and psoriatic arthritis (PsA) patients. **A,** One-dimensional ^1^H NMR spectra of urine samples obtained 12 weeks after treatment with infliximab (open circles) or etanercept (solid circles) from RA and PsA patients with a good response to treatment were subjected to supervised analysis (PLS-DA). The score in LV1 is plotted against the score in LV2, and the percent values on the x and y axes show the proportions of the total variance in the data captured by LV1 and LV2 in the PLS-DA model. The horizontal dashed lines at zero segregate samples with positive and negative scores. **B,** Weightings plot of the PLS-DA model of spectral data from urine samples obtained after treatment with infliximab or etanercept from RA and PsA patients with a good response at 12 months highlights major regions of the spectra that distinguish between the sample groups. The major discriminating peaks are labeled with their chemical shift (in parts per million) on the x-axis and LV weighting on the y-axis. The percent value on the y-axis indicates the proportion of the total variance in the data captured by LV1. See Figure 1 for other definitions.

Combining RA and PsA patients with a good response, we assessed which urinary metabolites changed after 12 weeks of treatment with infliximab and with etanercept using supervised PLS-DA analysis (sensitivity of 84.6% and specificity of 55.6%) (Figure 3) and GALGO analysis (sensitivity of 86.2% and specificity of 100%). Using the weightings plot, we found that increases in hippuric acid, citrate, and lactic acid were seen with infliximab treatment and increases in choline, phenylacetic acid, urea, creatine, and methylamine were seen with etanercept treatment. Due to the small patient numbers, we could not investigate the effects of etanercept and infliximab in RA and PsA separately.

## DISCUSSION

There were clear differences in the metabolic profiles of baseline urine samples of patients with RA who responded well to anti-TNF therapy compared with those who did not. This difference may be important as a novel predictor of responses to TNF antagonists. We have used 3 different data analysis methods to predict response, and each found that similar metabolites contributed. We have used GALGO analysis as well as PLS-DA analysis as it has been shown that genetic algorithms optimize the results by removing irrelevant variables, which dramatically improves the classification ability of models (34). All 3 methods identified histamine, glutamine, xanthurenic acid, and ethanolamine, while both PLS-DA and partial least-squares regression identified creatinine, p-hydroxyphenylpyruvic acid, and phosphocreatine, and both PLS-DA and GALGO identified phenylacetic acid and xanthine. Histamine, glutamine, phenylacetic acid, xanthine, xanthurenic acid, and creatinine were up-regulated in the urine samples from the patients who had a good response to therapy, while ethanolamine, p-hydroxyphenylpyruvic acid, and phosphocreatine were down-regulated.

One metabolite we identified as a strong discriminator in baseline urinary metabolites was histamine. Urinary histamine metabolites have also been suggested as a marker of disease activity in IBD (35), suggesting that it may be a generic marker of inflammatory processes. Histamine is most obviously associated with mast cell–dependent processes such as allergy, and histamine has been identified as a constituent of synovial fluid in arthritis (36). Histologic examination of synovial infiltrates in early RA has shown mast cells to be present (37), suggesting that these cells could be the source of the discriminating histamine. However, an alternative but significant route for histamine generation is via histidine degradation. Histamine arises in many tissues by the decarboxylation of histidine (38). It has long been known that TNFα promotes cachexia associated with chronic inflammatory disease, and this cytokine is known to have direct effects in accelerating muscle breakdown, leading to the release of free amino acids including histidine (39). Consistent with this, levels of histidine have been shown to be considerably higher in patients with RA and patients with systemic lupus erythematosus (40) compared to levels in controls.

Several of the other metabolites that we observed were also associated with catabolic processes and tissue degradation; for example, glutamine, xanthurenic acid, and ethanolamine can result from tryptophan and other amino acid degradation pathways. Tryptophan has been shown to be down-regulated in the plasma of patients with ankylosing spondylitis (AS) compared with the plasma of controls (41). The release of tryptophan from its binding serum protein has been shown to correlate with improvement in disease activity in AS (41), and this may be the same in RA.

A previous metabolomic study has suggested that alterations in serum levels of amino acids may be a useful marker of the presence and severity of osteoarthritis in the knee (42). The urine markers that we found either may be indicators of joint-specific degradation processes or may result from the systemic muscle and tissue changes associated with chronic disease, many of which are mediated through TNFα.

Previous work has investigated predictors of response to TNFα antagonists. Analysis of patients in the British Society for Rheumatology Biologics Register found that treatment with methotrexate or nonsteroidal antiinflammatory drugs (NSAIDs) predicted response to TNF antagonists (43). All the patients in our study were receiving methotrexate, and there were an equal number of patients receiving NSAIDs who had a good response compared to those who did not. Smoking has been associated with a poor response to infliximab (43), but only 1 of our patients smoked. Another group has found that the presence of RF or anti-CCP antibodies is associated with a reduced response to TNF antagonists (44), but all of our RA patients were positive for RF and/or anti-CCP antibodies. Baseline levels of TNFα may predict the dose of infliximab needed for optimal response (45), and other work has demonstrated that a combination of blood cytokines and autoantibodies can predict responses to etanercept (46). In our cohort there was a significant difference between CRP levels in patients who responded to TNF antagonists and those who did not. However, the partial least-squares regression analysis failed to find an association between CRP and baseline metabolites, suggesting that the association between baseline metabolites and response is independent of CRP.

Infliximab and etanercept alter metabolites in the urine differently, as there are clear differences in the metabolites at 12 weeks posttreatment. Increases in the metabolites hippuric acid, citrate, and lactic acid were associated with infliximab treatment, and increases in the metabolites choline, phenylacetic acid, urea, creatine, and methylamine were associated with etanercept treatment. The presence of choline suggests that etanercept may alter lipid metabolism.

We have also shown that the same metabolites are altered in the urine samples from patients with RA and PsA who responded to TNF antagonists. It may therefore be that chronic inflammatory diseases respond by a common mechanism to TNF antagonists.

This is the first demonstration that metabolomic techniques using 1-D NMR spectra can predict outcome to anti-TNF therapy in patients with severe RA, providing a sensitivity and specificity for response that has potential clinical utility despite a small initial cohort of patients. Our findings are verified by repeat analysis using alternative statistical techniques. There is a pressing need to confirm and extend these findings in a larger cohort of patients, combining metabolomic analyses with CRP and cytokine and autoantibody analyses to develop tests that can predict response without the need for empirical treatment, bringing closer the era of individually tailored therapy.

## AUTHOR CONTRIBUTIONS

All authors were involved in drafting the article or revising it critically for important intellectual content, and all authors approved the final version to be published. Dr. Young had full access to all of the data in the study and takes responsibility for the integrity of the data and the accuracy of the data analysis.

**Study conception and design.** Kapoor, Filer, Fisher, Taylor, Buckley, McInnes, Raza, Young.

**Acquisition of data.** Kapoor, Filer, Fisher, Taylor, Buckley, McInnes, Young.

**Analysis and interpretation of data.** Kapoor, Filer, Fitzpatrick, Fisher, Raza, Young.

## ROLE OF THE STUDY SPONSOR

Merck/MSD had no role in the study design or in the collection, analysis, or interpretation of the data, the writing of the manuscript, or the decision to submit the manuscript for publication. Publication of this article was not contingent upon approval by Merck/MSD.
